# Heterosis of endophytic microbiomes in hybrid rice varieties improves seed germination

**DOI:** 10.1128/msystems.00004-24

**Published:** 2024-04-09

**Authors:** Yuanhui Liu, Kankan Zhao, Erinne Stirling, Xiaolin Wang, Zhenyu Gao, Bin Ma, Chunmei Xu, Song Chen, Guang Chu, Xiufu Zhang, Danying Wang

**Affiliations:** 1 State Key Laboratory of Rice Biology and Breeding, China National Rice Research Institute, Hangzhou, Zhejiang, China; 2 Zhejiang Provincial Key Laboratory of Agricultural Resources and Environment, College of Environmental and Resource Sciences, Zhejiang University, Hangzhou, Zhejiang, China; 3 Agriculture and Food, Commonwealth Scientific and Industrial Research Organization, Adelaide, Australia; 4 School of Biological Sciences, The University of Adelaide, Adelaide, Australia; 5 The State Key Laboratory for Conservation and Utilization of Subtropical Agro-bioresources, South China Agricultural University, Guangzhou, Guangdong, China; University of Dundee, Dundee, Scotland, United Kingdom

**Keywords:** Rice (*Oryza sativa*), seed endophytic microbiota, heterosis, seed germination, high-throughput sequencing

## Abstract

Seed endophytic microbiomes are shaped by host and environmental factors and play a crucial role in their host growth and health. Studies have demonstrated that host genotype, including hybridization, affects seed microbiomes. Heterosis features are also observed in root-associated microbiomes. It remains unclear, however, whether heterosis exists in seed endophytic microbiomes and whether hybrid microbiota provide noticeable advantages to host plant growth, especially to seed germination. Here, we investigated the structure of seed endophytic bacterial and fungal communities from three hybrid rice varieties and their respective parents using amplicon sequencing targeting 16S rRNA and ITS2 genes. Heterosis was found in diversity and composition of seed endophytic microbiomes in hybrids, which hosted more diverse communities and significantly higher abundances of plant growth-promoting taxa, such as *Pseudomonas* and *Rhizobium* genera compared with their parental lines. Co-occurrence network analysis revealed that there are potentially tighter microbial interactions in the hybrid seeds compared with their parent seeds. Finally, inoculation of seed-cultivable endophytes, isolated from hybrids, resulted in a greater promotion of seed germination compared with those isolated from parent lines. These findings suggest that heterosis exists not only in plant traits but also in seed endophytic microbiota, the latter in turn promotes seed germination, which offers valuable guidance for microbiome-assisted rice breeding.

**IMPORTANCE:**

Genetic and physiological changes associated with plant hybridization have been studied for many crop species. Still, little is known about the impact of hybridization on the seed microbiota. In this study, we indicate that hybridization has a significant impact on the endophytic bacterial and fungal communities in rice seeds. The seed endophytic microbiomes of hybrids displayed distinct characteristics from those of their parental lines and exhibited potential heterosis features. Furthermore, the inoculation of seed-cultivable endophytes isolated from hybrids exhibited a greater promotion effect on seed germination compared with those isolated from the parents. Our findings make a valuable contribution to the emerging field of microbiome-assisted plant breeding, highlighting the potential for a targeted approach that aims to achieve not only desired plant traits but also plant-beneficial microbial communities on the seeds.

## INTRODUCTION

Plants and their associated microbiota act as a singular functional entity referred to as the “holobiont” (1, 2). All plant compartments provide microhabitats that support diverse microorganisms in regulating the fitness and resilience of the holobiont in response to environmental perturbations (2). Seeds represent a remarkable stage in the life cycle of spermatophytes and a distinct niche for a unique microbiota that harbors various adaptations for successful colonization (3). The seed microbiome’s existence and function went unnoticed for decades (4, 5), despite early suggestions of microorganisms in seeds primarily being focused on pathogenic fungi. Currently, it is evident that seed microbiomes are highly diverse, encompassing as many as 9,000 microbial species, many of which play a crucial role in fundamental physiological processes, including seed dormancy and germination, environmental adaptation, disease resistance and tolerance, and growth promotion (6, 7). However, compared with other plant compartments, such as rhizosphere and phyllosphere, research on the seed microbiome remains relatively limited.

Seed endophytes are particularly intriguing among seed-associated microbiomes because they escape competition with soil microorganisms, are in intimate contact with the plant tissues from an early stage, and play a critical role in transmitting potential beneficial and pathogenic microorganisms from one generation to another (8, 9). The assembly of seed endophytic microbiomes is expected to be influenced by intrinsic host genotypic traits, lifestyle, and external environmental conditions (10, 11). For instance, seeds from eight native plant species grown under the same environmental conditions hosted unique microbial communities indicating that host genotype was the main driver of seed microbiome (10). The host genetics of parental lines may play a crucial role in determining the seed phenotype, affecting features such as seed anatomy, immune response, and chemical composition. These characteristics, in turn, can influence the assembly of the seed microbiome (12, 13). In addition to plant genotype, several environmental factors such as climatic conditions and soil characteristics can affect seed endophytic microbiomes (14).

Rice (*Oryza sativa*) represents one of the most important staple crops in the world. The utilization of heterosis in rice is a crucial element in enhancing crop quality and productivity (15). Heterosis, or hybrid vigor, refers to the phenomenon where the offspring of two genetically diverse parents exhibit traits that are superior to those of both parents (16). This approach has been widely employed to enhance the yield and quality of major cereal crops as well as commercial varieties of vegetable and flower crops (17, 18). Recent research demonstrates that long-term plant breeding not only shapes plant characteristics but also has a significant impact on plant-associated microbiota (19–21). Hybrid maize rhizosphere and leaf microbiota and hybrid rice root microbiota exhibit heterosis as seen in their community diversity and composition when compared with their respective parental lines (22, 23). The impact of breeding on microbial communities is more pronounced in seeds compared with other plant compartments (24). Several pioneering studies have indicated that seed endophytic microbiomes in hybrids are distinct from their parental lines across a variety of plants, including Styrian oil pumpkin (24), rice (25), and maize (26). However, it remains largely unknown whether the observed differences in microbiome composition between hybrids and inbred varieties contribute to plant trait heterosis, such as promoting germination.

To investigate the effects of hybridization on rice seed endophytic microbiomes and the consequential contribution of seed endophytic microbiomes to seed germination, we used amplicon sequencing to determine seed endophytic bacterial and fungal communities from three rice hybrids and their respective parental lines. These plants were grown together in the same experimental fields to control for environmental variables. We also conducted an inoculation and germination experiment to determine the impact of seed endophyte extracts from both hybrids and parents on germination phenotypes. Our findings suggest that the seed endophytic microbiomes of hybrids exhibit potential heterosis features and superior effects in promoting seed germination. Our study establishes a link between hybrid heterosis in their seed microbiota and their potential beneficial function in promoting plant growth. This represents a critical step toward microbiome-driven breeding for plant-beneficial microbes.

## RESULTS

### Seed endophytic microbiome of hybrid rice exhibits potential heterosis feature

**Fig 1:**
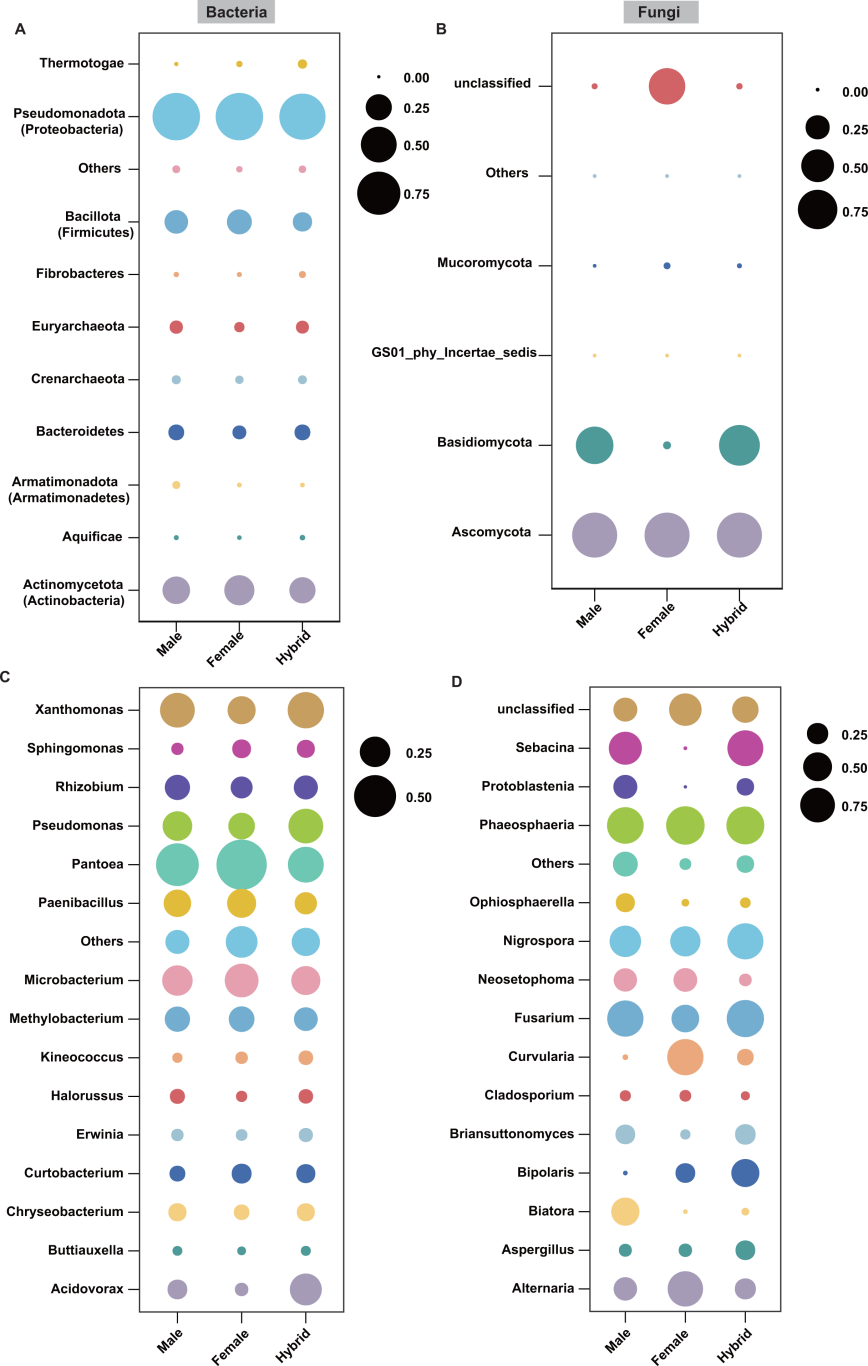
The bacterial and fungal communities’ composition in the seeds of hybrid varieties and their parental lines. Bubble plots showing the relative abundances of bacterial (**A, C**) and fungal (**B, D**) taxa at the phylum and genus level in the seeds of hybrid varieties (*n* = 15), male (*n* = 15), and female parental lines (*n* = 15). Due to the updated bacterial classification names in 2021, the names in figure A have been revised to reflect the updated nomenclature, whereas the old names are provided in parentheses. The size of the bubble represents the relative abundances of the microbial community.

To investigate whether hybrids differed from parental lines in seed endophytic bacterial and fungal diversity and composition, we planted three widely cultivated hybrid rice varieties with heterosis in yield, along with their parental lines, in a paddy field. The seed endophytic microbiome analysis was conducted on mature seeds using high-throughput sequencing of the bacterial 16S rDNA gene V5-V7 region and the fungal ITS2 region. After quality filtering and removal of chimeras, a total of 3,262,205 bacterial sequences (an average of 72,493/sample) and 6,177,900 (an average of 137,286/sample) fungal sequences were retained. Following the exclusion of chloroplast and mitochondrial sequences, 1,721 bacterial ZOTUs and 422 fungal ZOTUs were identified. Taxonomic classification was conducted for each hybrid rice combination, revealing that, aside from specific microbial taxa, major phyla and genera exhibited consistent trends in relative abundance differences among male and female parents and hybrids within each combination (Fig. S1). Consequently, we collectively analyzed these three hybrid rice combinations to identify shared patterns in differences between hybrids and parental lines. The dominant bacterial phyla were Pseudomonadota (Proteobacteria) and Actinomycetota (Actinobacteria), accounting for an average of 75.9% and 16.3%, respectively (Fig. 1A). The genera of *Pantoea* (27.7%), *Xanthomonas* (12.4%), *Pseudomonas* (12.0%), and *Microbacterium* (11.0%) were found to be abundant in the seeds of hybrids and parents (Fig. 1C). Fungal communities were dominated by the phyla of Ascomycota (92.7%) and Basidiomycota (4.8%) (Fig. 1B), as well as dominated by the genera of *Phaeosphaeria* (25.9%) and *Nigrospora* (18.2%) (Fig. 1D).

**Fig 2:**
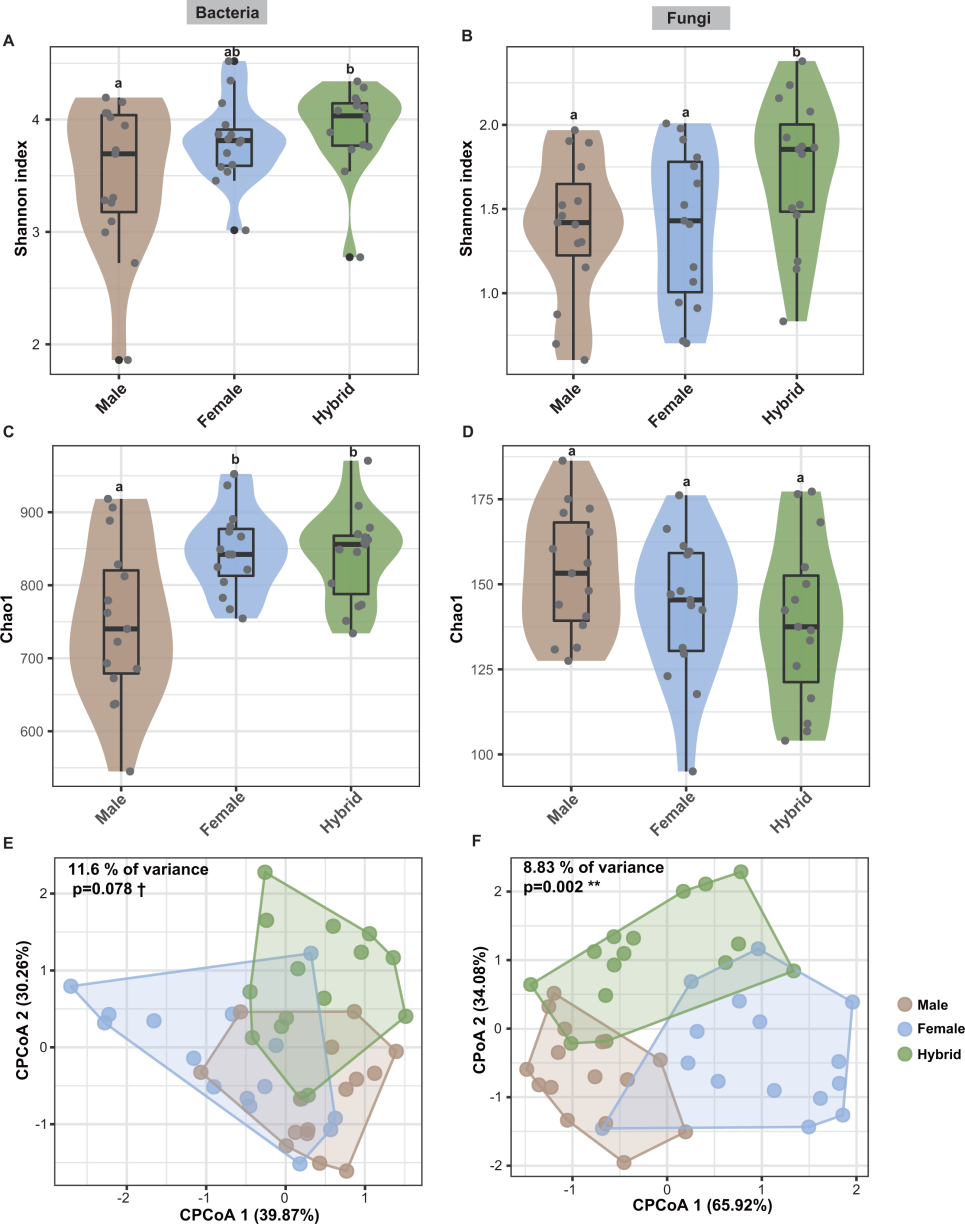
The bacterial and fungal communities’ diversity in the seeds of hybrid varieties and their parental lines. Alpha diversity of bacterial (**A, C**) and fungal (**B, D**) communities as determined by Shannon and Chao1 indices; different lowercase letters indicate significant difference (*P* < 0.05; One-way ANOVA and Dunn’s multiple-comparison test). Constrained principal coordinates analysis (CPCoA) based on Bray-Curtis distance are plotted for bacterial (**E**) and fungal (**F**) communities in the seeds of hybrid varieties (*n* = 15), male (*n* = 15), and female parental lines (*n* = 15). ANOVA-like permutation tests were performed to assess statistical significance of the constrained axis. Significance levels of each symbol are **P ≤ 0.01, *P ≤ 0.05, and †*P* ≤ 0.10.

It was recently suggested that the microbiota exhibits extended plant traits with heterotic features in maize hybrids, based on an analysis of microbial composition and diversity, and microbiota heterosis is thus defined as the presence of hybrid microbial trait values that are higher or lower than those of both parents (22). The microbiomes of hybrid rice manifest a potential heterosis feature in alpha diversity. The Shannon (representing community richness and evenness) and Chao1 (indicating community richness) indices were both significantly higher for the bacterial community in hybrids compared with their parents (Fig. 2A and C; Fig. S2A and C). In contrast, the fungal communities present in hybrids demonstrated significantly higher Shannon diversity compared with both parents, except in combination C3 (*P* < 0.05) (Fig. 2B; Fig. S2B). However, there was no significant difference in Chao1 index among these genotypes (Fig. 2D; Fig. S2D). Constrained principal coordinate analysis (CPCoA) based on Bray-Curtis distance exhibited a distinct segregation of fungal communities (*P* = 0.002, *n* = 45) while showing a comparatively milder distinction in bacterial communities (*P* = 0.078, *n* = 45) between hybrids and parental lines. This analysis accounted for 11.6% and 8.83% of variations in bacterial and fungal communities, respectively (Fig. 2E and F).

### The hybrid rice seeds enrich higher abundances of potential beneficial taxa

**Fig 3:**
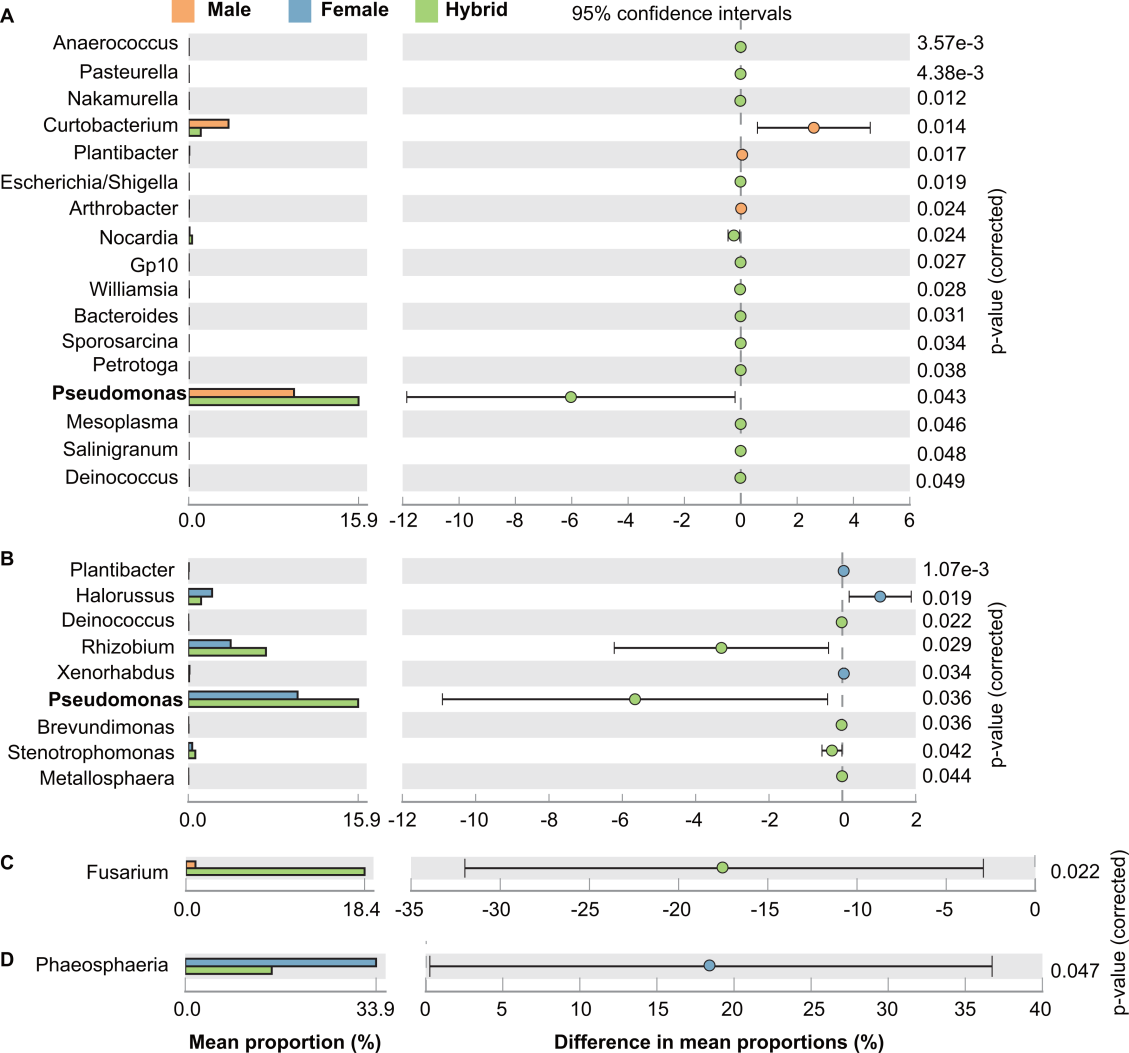
Variation analysis of endophytic microbiomes at the genus level in hybrid seeds in comparison to male parent seeds or female parent seeds. (**A**) and (**B**) represent bacterial communities in hybrid (*n* = 15) compared with the male parent (*n* = 15) and female parent (*n* = 15), respectively. (**C**) and (**D**) represent fungal communities in hybrid compared with the male parent and female parent, respectively.

The statistical analysis of taxonomic and functional profiles (STAMP) method was performed to identify the differences in taxonomic abundances between the hybrids and parental lines at genus level. The relative abundances of 17 bacterial genera exhibited significant differences between the hybrid and the male parent, whereas nine bacterial genera showed significant distinctions between the hybrid and the female parent (Fig. 3A and B; *P* < 0.05). A majority of the differentially abundant genera, including *Rhizobium*, *Pseudomonas*, and *Stenotrophomonas*, were enriched in the hybrid seeds (Fig. 3A). Specifically, *Pseudomonas* in hybrids increased by 6.03% (male) and 5.66% (female) (*P* < 0.05) with a significant enrichment (Fig. 3A and B). Only two genera exhibited a disparity in abundance when comparing the fungal communities of hybrids and parental lines. *Fusarium* had a significantly higher relative abundance in hybrids when compared with male parents, whereas *Phaeosphaeria* was significantly enriched in female parents relative to hybrids (Fig. 3C and D).

**Fig 4:**
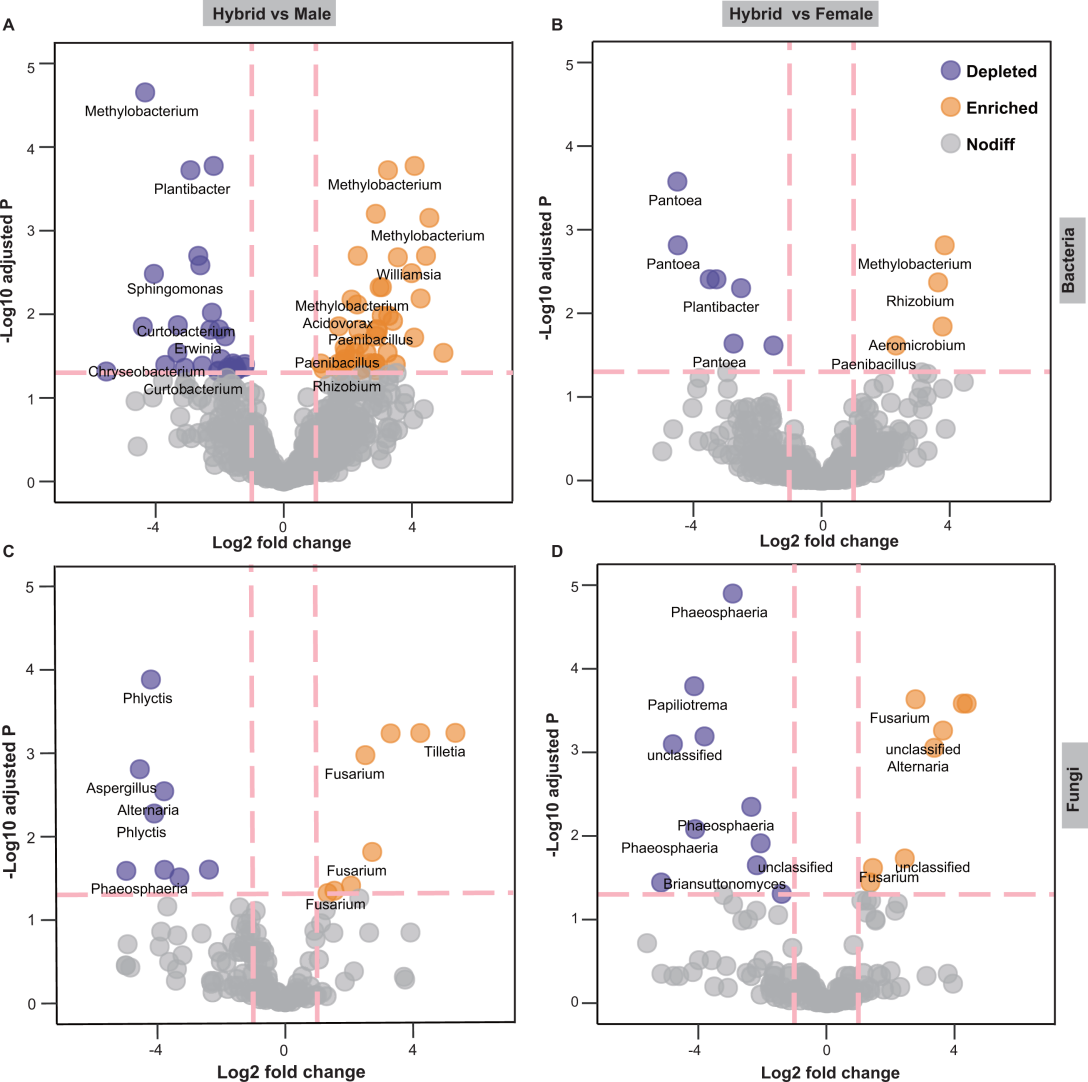
Volcano plots showing differential ZOTUs of seed endophytic bacterial (**A, B**) and fungal (**C, D**) communities in hybrid varieties (*n* = 15), male (*n* = 15), and female parental lines (*n* = 15). Variety X vs variety Y (e.g., hybrid vs male) represents the significantly differential ZOTUs in hybrid varieties relative to the male parent (*P* < 0.05); yellow points indicate significantly enriched ZOTUs, and purple points indicate significant depleted ZOTUs (*P* < 0.05).

The numbers of enriched/depleted ZOTUs were higher in hybrids vs male parents than in hybrids vs female parents. There was a larger proportion of enriched ZOTUs than depleted ZOTUs (49 vs 29) in hybrids relative to their male parent, which were mainly composed of the genera *Methylobacterium* and *Paenibacillus* (Fig. 4A). Compared with the female parent, hybrids were significantly enriched in 4 ZOTUs, which mainly belonged to *Rhizobium*, *Pseudomonas*, and *Paenibacillus*, and significantly depleted in 7 ZOTUs that were mainly composed of *Pantoea* (Fig. 4B). For fungal communities, the hybrids exhibited a significant enrichment in 10 ZOTUs, predominantly composed of *Fusarium* compared with male and female parents (Fig. 4C and D).

### Co-occurrence networks in hybrid rice are tighter than those in their parental lines

**Fig 5:**
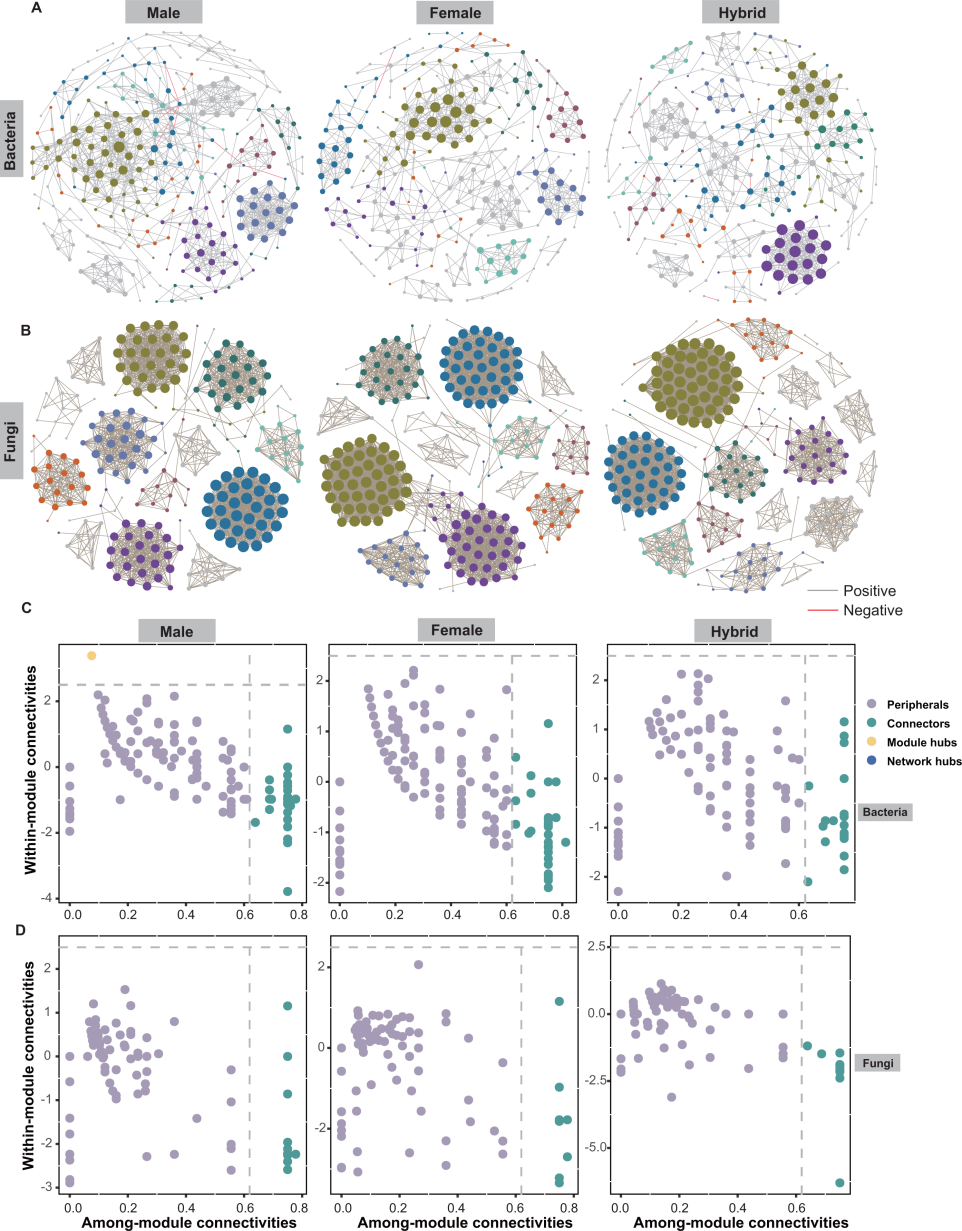
Co-occurrence networks and the distribution of ZOTUs in bacterial and fungal networks in rice of different genotypes. Co-occurrence networks of bacterial (**A**) and fungal (**B**) communities found in hybrid varieties (*n* = 15), male (*n* = 15), and female parental lines (*n* = 15). Each variety’s network was constructed based on Spearman’s correlation coefficients (ρ) of >0.8 or < −0.8 and false discovery rate-corrected *P*-values  <  0.001. Node size is proportional to relative abundance, and node colors indicate modules. *Zi-Pi* plot showing the distribution of OTUs based on their topological roles in bacterial (**C**) and fungal (**D**) networks in rice of different genotypes. The threshold values of *Zi* and *Pi* for categorizing OTUs were 2.5 and 0.62, respectively.

To further compare the interactions and associations of endophytic microbiomes in hybrids and parental lines, we assessed the co-occurrence patterns of bacterial and fungal communities based on the relative abundances of the top 300 ZOTUs. Network complexity and stability, as indicated by node and edge number, average degree, and connectance, were higher in the fungal network than in the bacterial network for each genotype. For bacterial networks, the number of nodes and edges, average degree, and connectance were highest in the male parent, medium in hybrid, and lowest in the female parent (Fig. 5A; Table S1). The higher clustering coefficient and number of clusters, coupled with the lower average path length, indicate a greater potential for bacterial interactions in the hybrid network compared with the parental lines (Table S1). In contrast, the fungal network’s topological properties, such as edge numbers, connectance, and average degree, displayed the highest values in the female parent, the lowest in the male parent, and intermediate values in the hybrids (Fig. 5B; Table S1). The hybrid network displayed a higher clustering coefficient and a greater number of clusters than those observed in the parental lines (Table S1).

To further elucidate the topological significance of each ZOTU within the networks, we characterized the within-module connectivity (*Zi*) and among-module connectivity (*Pi*). Within the male parent bacterial network, a total of 48 connectors were identified, with affiliations to *Methylobacterium* (25%), *Sphingomonas* (12.5%), and *Pseudomonas* (8.3%) (Fig. 5C). Additionally, a module hub affiliated with *Rhizobium* was also found in the network. In the female parent and hybrid networks, a total of 50 and 48 connectors were identified, respectively (Fig. 5C). These connectors were mainly composed of *Pseudomonas* (18% and 14.5%), *Methylobacterium* (12% and 14.5%), and *Sphingomonas* (10% and 12.5%). In the case of fungal networks, the hybrids and female networks had 14 and 9 connectors, respectively, with *Phlyctis* being the predominant category in both (28.5% and 22.2%) (Fig. 5D).

### The effect of seed endophytes on the germination phenotypes

To compare the effect of the seed endophytic bacterial communities of hybrid and the parental lines on seed germination, we conducted germination assays for each variety using cultivable seed endophyte suspensions (SES) obtained from both the hybrid and corresponding parental lines. Cultivable seed endophytic bacteria isolated from each variety underwent DNA extraction, followed by the characterization of bacterial communities through amplicon sequencing targeting the V5-V7 variable regions of the 16S rRNA gene. Following quality filtering and chimera removal, a total of 4,432,906 bacterial sequences (averaging 164,181 per sample) were retained. Subsequent exclusion of chloroplast and mitochondrial sequences led to the identification of 547 bacterial ZOTUs. Similar to the endophytic bacterial community in seeds, taxonomic classification indicates that the culturable bacterial community is predominantly composed of the phylum Pseudomonadota (76.7%) and the genera *Pseudomonas* (15.7%), with a notable and statistically significant enrichment of *Pseudomonas* in hybrids (Fig. S3A and B). Additionally, the Shannon indices of seed culturable endophytic bacterial communities showed a significant increase in hybrids compared with their parent varieties (Fig. S3C).

**Fig 6:**
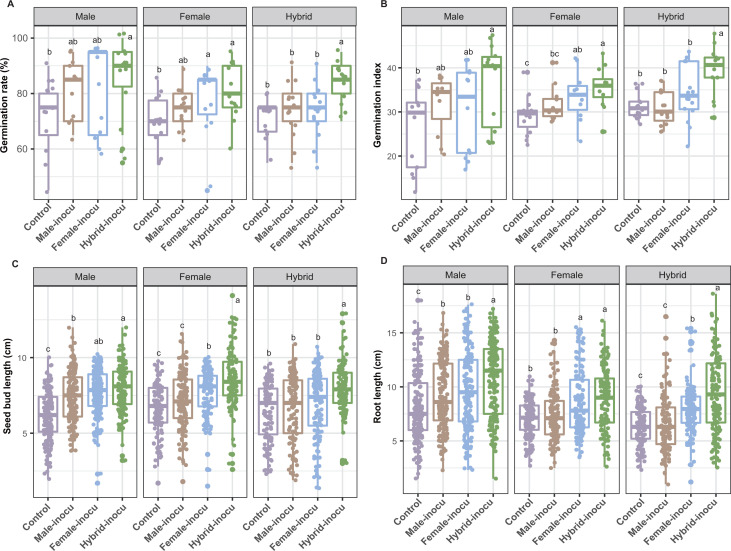
The germination phenotypes, including the germination rate, germination index, and the bud and root length of seeds inoculated with seed endophytic microbiome suspensions. Different lowercase letters indicate a significant difference (*P* < 0.05; One-way ANOVA and Dunn’s multiple-comparison test). Control: sterile phosphate-buffered saline (PBS) solution; Male-inocu, Female-inocu, and Hybrid-inocu represent seed endophytic microbiome suspensions enriched from male, female, and hybrid seeds, respectively.

The germination phenotypes of seeds were analyzed, including the germination rate, germination index, seed bud length, root length, and dry weight of bud and root. The findings revealed that hybrid seeds inoculated with SES derived from hybrids displayed notably superior germination phenotypes. This superiority was evident in various aspects, such as germination rate, germination index, as well as length of seed bud and root, surpassing those observed in other treatments (Fig. 6A through D; Fig. S4; *P* < 0.05). In the case of the male parent, seeds inoculated with SES obtained from hybrids exhibited a notably higher root length compared with other treatments (Fig. 6D). However, the germination rate, germination index, seed bud length, and seed bud and root dry weight of seeds postinoculation with SES from the female parent were lower than that from hybrid, and the promotion of germination showed a similar efficacy (Fig. 6A through C; Fig. S4 and S5). For the female parent, aside from the notable increase in seed bud length and bud dry weight observed in seeds inoculated with SES obtained from hybrids compared with other treatments (Fig. 6C; Fig. S4 and S5A; *P* < 0.05), the germination rate, germination index, and root length were all significantly higher in seeds inoculated with SES obtained from both hybrids and the female parent, in comparison to other treatments (Fig. 6A, B and D; Fig. S4 and S5A; *P* < 0.05). Root dry weight was observed to be higher in seeds inoculated with SES from hybrids compared with other treatments, although the difference was not statistically significant (Fig. S5B).

## DISCUSSION

**Fig 7:**
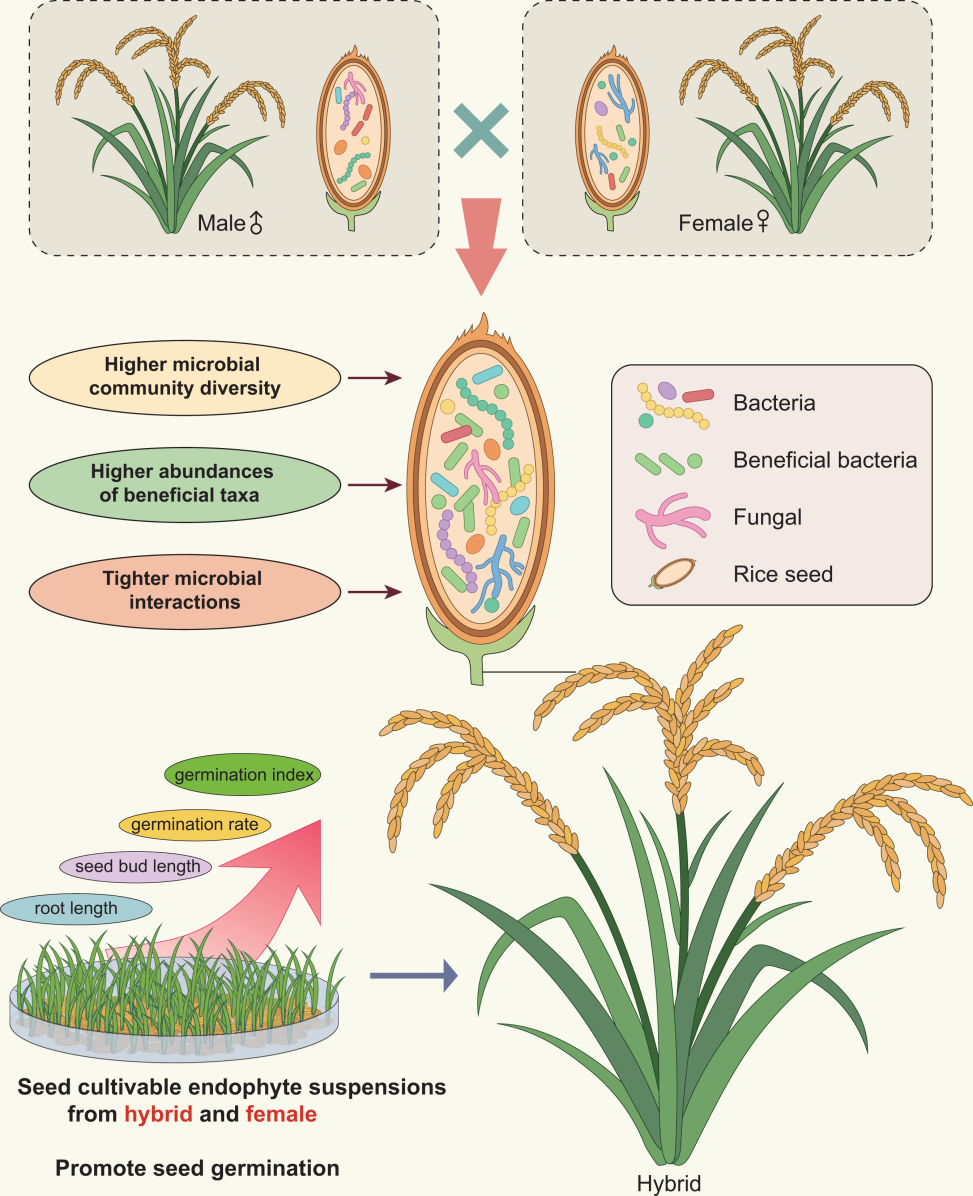
The seed endophytic microbiomes of hybrids display distinct characteristics from those of their parental lines and exhibit potential heterosis features.

The role of host genotype in shaping microbiome composition and function is a crucial topic for both basic and applied research into plant-associated microbial communities. Heterosis (hybrid vigor) is a natural genetic phenomenon in which cross-pollinated hybrids display superior phenotypic performance compared with their parents. This enhanced performance includes traits such as biomass, speed of development, fertility, and disease resistance (16, 27). It was recently suggested that the microbiota may be an extended plant trait displaying heterotic features (22). In this study, we aimed to determine if hybrid rice seed endophytic microbiomes also displayed heterosis-like traits by analyzing the seed endophytic bacterial and fungal communities from three hybrid rice varieties and their parental lines. We demonstrated that seed endophytic microbiomes of hybrid rice differ reliably from their parental lines, with a higher microbial diversity (Fig. 7). The impact of hybridization on the diversity of the rhizosphere microbiome was just recently identified (19, 20, 22, 23). Genotype-specific seed microbial communities have been previously studied and identified in several plant species (28–30). Notably, there are significant differences in the diversity of seed endophytic microbes observed between hybrid and parental lines in rice, maize, and Styrian oil pumpkin (24–26). In this study, host genotypes accounted for 11.6% and 8.83% of the overall variation in bacterial and fungal microbiome composition, respectively. This is a high proportion relative to other studies of host genetic microbiome control (22, 31). During the process of breeding, the genetic traits of plants are selected, which possibly also change the microbiome, which is why a genotype-specific seed and rhizosphere microbiome can be observed (10, 29).

In general, Pseudomonadota (Proteobacteria), particularly γ-Pseudomonadota, are the most predominant seed endophytic bacteria isolated from various plant species, with *Actinomycetota* (*Actinobacteria*) and *Bacillota* (*Firmicutes*) also well represented (8, 32). *Ascomycete* and *Basidiomycete* are two common fungal phyla that are frequently associated with seeds (33, 34). Similarly, our study revealed that the dominant phyla of bacteria and fungi in seeds were *Pseudomonadota* (*Proteobacteria*) and *Ascomycota*. The impact of hybridization was more pronounced in the bacterial community compared with the fungal community, as evidenced by the greater differences observed between hybrids and parental lines in bacterial communities as opposed to fungal communities. One possible explanation is that bacterial communities associated with seeds are mainly impacted by genotypes, whereas fungal communities are predominantly influenced by local environmental conditions and non-host genotypes (24, 35).

Differential analysis of taxonomic abundances revealed that certain distinguishing taxa between hybrids and their parental lines have been associated with beneficial effects on plant growth and health. For example, hybrid seeds harbored higher abundances of *Pseudomonas*, *Rhizobium*, and *Fusarium* genera compared with parental lines. *Pseudomonas* is a major group of bacteria commonly observed as plant growth-promoting bacteria (36–38). In particular, it has been discovered that this genus is the dominant seed-associated taxon among several plant hosts and exhibits a remarkable capacity for siderophore production (38). *Rhizobium*, the root-nodule endosymbionts of leguminous plants, also forms endophytic associations with roots and seeds of rice (39, 40). A significant increase in biomass and yield has also been observed in greenhouse-grown rice plants inoculated with *Rhizobium leguminosarum* bv. *phaseoli* (41). Previous research has demonstrated that epiphytic fungi belonging to the *Fusarium* genus can improve seed germination in the rainy tropics (42).

Co-occurrence patterns are widely observed and play crucial roles in comprehending the structure of microbial communities (43). Our study found that the complexity and stability of fungal networks were higher than bacterial networks across all genotypes. This observation may be attributed to the greater resistance of fungal interactions to potential environmental disturbances compared with bacteria. This finding is consistent with the observation of a less stable bacterial network compared with the fungal network under drought stress (44). Furthermore, we discovered that hybridization has an impact on the structure of co-occurrence networks, not only in bacteria but also in fungi. Specifically, we observed a greater level of connectivity in the hybrid networks when compared with the parental lines. This finding is consistent with a previous study that suggested the domestication of host plants, involving alterations in plant genomes and changes in the relationship with microbiomes, can impact the structure of microbial co-occurrence networks in rice seeds (45). In bacterial networks, the most prevalent bacterial connectors consist mainly of genera such as *Methylobacterium*, *Sphingomonas*, and *Pseudomonas*, which are known to be beneficial for plant growth and health (38, 46).

It is difficult to determine if the observed differences in seed microbiomes between hybrids and their parents can reliably indicate differences in microbiome function and/or seed germination. To further explore the impact of host genotype on the seed endophytic microbiomes and whether they feed back and contribute to the germination of seeds, we extracted suspensions of cultivable endophytic bacterial community and inoculated them into the seeds of each rice variety, then conducted a germination experiment. Here, we found that the germination phenotypes, including germination rate, germination index, seed bud length, and root length, were significantly higher in seeds inoculated with SES obtained from hybrid compared with other treatments. This study has confirmed that seed endophytic microbiomes in hybrids not only exhibit heterosis features but also contribute to the mechanism of hybrid vigor in the host (Fig. 7). Thus far, the expanding body of literature has demonstrated that seed endophytes exhibit promising plant growth promoting activities. It has been observed that the elimination of microbiota from seeds results in a notable decrease in the germination and development of rice, soybeans, beans, and maize (47–49). A synthetic bacterial community derived from the natural microbiota of maize seeds demonstrated the ability to restore germination and promote seedling growth upon reintroduction into disinfected seeds (49). Collectively, these studies indicate that the germination and growth rates of seedlings can be positively influenced, at least in part, by the bacteriome associated with seeds. Seed bacterial endophytes isolated from rice also exhibited plant growth-promoting activities including hormone modulation, nitrogen fixation, siderophore production, and phosphate solubilization (38). The cultivable seed endophytic bacterial community in each variety was characterized using high-throughput sequencing of the bacterial 16S rDNA gene V5-V7 region. The findings unveiled a higher diversity of cultivable seed endophytes in hybrids, accompanied by a significant enrichment of potential growth-promoting bacteria. This heightened diversity and enrichment may contribute to the pronounced effect of promoting germination. However, further investigation is needed to elucidate the specific mechanisms through which these microorganisms facilitate seed germination. For instance, the endophytic bacteria associated with *Ammodendron* bifolium enhanced the efficiency of endosperm utilization, leading to a more pronounced degradation of sucrose, protein, and triglycerides within the seed (50). Furthermore, these bacteria were found to produce hydrolytic enzymes, further contributing to the metabolic processes involved (50). Similarly, Verma and colleagues revealed that seed bacteria have the capability to express the enzyme amylase, thereby enhancing the efficiency of endosperm utilization during both germination and seedling growth (51). Interestingly, the structure of the seed microbial community and its promotion effect on seed germination were found to be similar between hybrids and female parents. This may be explained by that seed microbiomes’ direct vertical transmission is more likely through the female parent than through pollen as rice florets are self-pollinated (52).

## MATERIALS AND METHODS

### Rice genotypes

Three elite rice hybrid varieties, Tianyouhuazhan (TFHZ), Zhongzheyou-H7 (ZYH7), and Huazheyou-261 (HZ261), which have been widely planted for their excellent heterosis characteristics such as high yield, good grain quality, and wide adaptability, were selected along with their parental lines (HZ♂×TF♀), (H7♂×ZZA♀), and (R261♂×HZA♀) (Table S2). For the three hybrid rice combinations (C1-C3), the hybrid seeds were obtained via a standard seed production process by the local seed production companies, with the parental seeds being supplied by researchers of China National Rice Research Institute (Table S2).

### Field experimental, rice seed sampling strategy and surface sterilization

Seeds were planted in a paddy field in Huteng village, Hangzhou, Zhejiang Province, China (30°4′ N, 119°5′ E) in June 2021. The basic characteristics of the soil were assessed using the methods described by Bao (2000) (53). Soil properties were as follows: pH 5.92 (soil:water = 1:2.5), available nitrogen (N) 148.1 mg kg^−1^, available phosphorous (P) 18.4 mg kg^−1^, available potassium (K) 150.3 mg kg^−1^, and organic matter 33.7 mg kg^−1^. The nine rice varieties were randomly arranged in the field with each treatment block containing 72 mounds spaced 25 cm × 20 cm apart. Each variety block was separated with an empty row. Pregerminated seeds were cultivated in a seedbed, and 25-day-old seedlings were transplanted into individual mounds with two seedlings planted per mound. During the experiment, N was applied as urea with a total rate of 195 kg ha^−1^ in three applications: the first application was applied a s basal fertilizer 1 day before transplantation (50% of total), the second was applied as tillering fertilizer 7 days after transplantation (30% of total), the third was applied as ear fertilizer at booting (20% of total). Phosphorus fertilizer was applied 1 day before transplanting as superphosphate Ca(H_2_PO_4_)_2_·H_2_O at a rate of 300 kg ha^−1^. Potassium fertilizer was applied as basal fertilizer (50%) and ear fertilizer (50%), with a total rate of 75 kg ha^−1^ as KCl.

Mature seeds of each variety were harvested, dried, and sealed in a sterilized nylon bag and stored at 4°C until further study. To minimize the impact of the seed surface microbiome, we selected five replicates for each variety, consisting of approximately 0.5 g of seeds, and subjected them to surface sterilization following the methods described by Liu et al. (54). The seeds were washed twice with sterile water and then treated with 70% (vol/vol) ethanol for 2 min. The ethanol was then discarded, and the seeds were washed again with sterile water to remove any residual ethanol. The seeds were immersed in a sterilization solution containing 2% vol/vol NaClO, 3% wt/vol NaCl, 0.1% wt/vol Na_2_CO_3_, and 0.15% wt/vol NaOH, and shaken (150 rpm) for 30 min. The seeds were washed seven times with sterile water and then dried in a sterile environment before being ground for further study. To validate the effectiveness of sterilization, 1 mL aliquots of the last-step washing water were plated on different growth agar media [Luria Bertami (LB), Mannitol Soya flour (MSF), and Potato Dextrose Agar (PDA)] and examined for microbial growth after incubation at 30°C for 7–15 days, with aliquots of washing water of unsterilized seeds as control (55) (Fig. S6).

### Rice seed endophytic inoculants affect germination phenotypes

To investigate the impact of seed endophytic bacterial communities on rice germination, cultivable seed endophyte suspension (SES) was inoculated onto the seeds of each rice variety and subsequently germinated according to the method described by Wagner et al. (56) with modifications. For each hybrid rice combination, we conducted a completely randomized design with two factors, in which we manipulated three plant genotypes (male, female, and hybrid) and four endophyte inoculants [SES enriched from female, male, hybrid, and sterile phosphate-buffered saline (PBS) solution as control]; each treatment had five replicates. The SES for each variety was prepared as follows: the surface-sterilized seeds were added to PBS solution and then homogenized by grinding. The resulting homogenates were then transferred to R_2_A liquid medium and cultured at 28°C with shaking (120 rpm) for 48 hours. The cultured SES was subsequently subjected to centrifugation to separate the supernatant, followed by two successive washes with PBS buffer to mitigate any potential disparities arising from variations in the chemical composition of the SES. The endophyte inoculant was standardized to a uniform concentration by measuring the absorbance value at 600 nm and adjusting it to an optical density (OD_600_) of 0.6 with a sterile PBS solution. Subsequently, the endophyte inoculant isolated from each variety underwent DNA extraction and characterization of the bacterial communities through amplicon sequencing targeting the V5-V7 variable regions of the 16S rRNA gene. The method is detailed in the section titled “DNA extraction, 16S rRNA gene, and ITS region sequencing” below.

Twenty surface-sterilized seeds were placed onto sterile quartz sand with adequate moisture in Petri dishes for each per genotype-inoculum combination and inoculated with 2 mL of endophyte inoculant. Petri dishes were then incubated in a growth chamber (Fisher Scientific, Inc., Suwanee, GA) at condition (28 ± 2°C, 12 h: 12 h photoperiod with 60% relative humidity) as described previously (57). We commenced daily observations from the initial day of cultivation. Employing the “broken breast white” criterion as the germination standard, we documented the daily count of germinated seeds and computed the germination rate and index (58) after 7 days. After 10 days, we measured seed bud length and root length using a digital scanner (Epson Perfection V800, Indonesia) and a Winrhizo PRO 2016 root analysis software application. Simultaneously, dry weights of bud and root were determined.

### DNA extraction, 16S rRNA gene, and ITS region sequencing

The surface-sterilized seeds for each variety were ground in a sterile mortar with liquid nitrogen after which the total DNA was extracted using a Fast DNA SPIN extraction kit (MP Biomedicals) following the manufacturer’s protocol. The purity and concentration of DNA samples were measured by agarose gel electrophoresis and a NanoDrop One Microvolume UV-Vis spectrophotometer (Thermo Fisher Scientific), respectively. The bacterial and fungal communities were assessed using a targeted amplicon strategy. The primer pairs (799 f: 5’- AACMGGATTAGATACCCKG-3′; 1193 r: 5′-ACGTCATCCCCACCTTCC-3′) targeting the 16S rRNA gene V5-V7 hypervariable regions and the primer pairs (ITS3-f: 5′-GCATCGATGAAGAACGCAGC-3′; ITS4-r: 5′-TCCTCCGCTTATTGATATGC-3′) targeting the ITS2 region were used. Polymerase Chain Reaction (PCR) amplification was carried out as follows: 94°C for 5 min; 30 cycles of denaturation at 94°C for 30 s, annealing at 52°C for 30 s, and extension at 72°C for 30 s; final extension was performed at 72°C for 10 min. The amplicon libraries were generated using the NEBNext Ultra DNA Library Prep Kit for Illumina (New England Biolabs, MA, USA) according to the manufacturer’s instructions, and index codes were added. The quality of the library was evaluated using a Qubit 4.0 Fluorometer (Thermo Fisher Scientific, MA, USA). The amplicon sequencing was carried out using an Illumina HiSeq PE250 sequencing platform (Guangdong Magigene Biotechnology Co., Ltd. Guangzhou, China).

### Processing of amplicon sequencing data and statistics

The paired-end sequence reads were subjected to a series of processing steps including merging, trimming, filtering, and generating into zero-radius operational taxonomic units (ZOTUs) using USEARCH (v11) with the UNOISE3 algorithm (59). Taxonomic classification was performed using SINTAX and the Ribosomal Database Project (RDP) and UNITE v9 databases as bacterial and fungal references, respectively.

Data analyses were performed using R version 4.0.5 (R Core Team, 2021). The analysis of the ZOTU table, feature table, and taxonomic information was conducted using *phyloseq* (60). Alpha diversity was determined using Shannon and Chao1indices with *vegan*. Normality was assessed using Shapiro tests. If the data were normally distributed, a one-way analysis of variance (ANOVA) was used, otherwise a Kruskal-Wallis test was used. Constrained principal coordinates analysis (CPCoA) based on Bray-Curtis distance was performed to determine the effect of different varieties of microbial community assembly, and an ANOVA-like permutation test was performed to assess statistical significance of the constrained factors. Co-occurrence network for each variety was constructed based on Spearman’s correlation coefficients (ρ) of >0.8 or < −0.8 and false discovery rate-corrected *P*-values  <  0.001 (61). The topological features of the networks were calculated using the *igraph* package in R (62). The network plots were visualized using the interactive *Gephi* 0.8.2. STAMP was used to identify significantly different species associated with rice varieties. The negative binomial model was utilized to investigate the relative abundance of microbial taxa that varied between hybrids and parents. The resulting *P*-values were subjected to the Benjamini and Hochberg False Discovery Rate (FDR) procedure for adjustment (63). ANOVA and Duncan’s multiple tests were employed to compare the germination rate, root length, and germinal length of rice plants inoculated with different endophyte suspensions. All graphs were generated using *ggplot2* (64) in R.

## Data Availability

The raw sequence data reported in this paper have been deposited in the Genome Sequence Archive in National Genomics Data Center, China National Center for Bioinformation/Beijing Institute of Genomics, Chinese Academy of Sciences (GSA: CRA010709). All scripts required for the computational analyses performed in this study are available at https://github.com/lyhca/hybrid-seed.

## SUPPLEMENTAL MATERIAL

Supplemental material msystems.00004-24-s0001.pdf Fig. S1-S6; Tables S1 and S2.

ASM does not own the copyrights to Supplemental Material that may be linked to, or accessed through, an article. The authors have granted ASM a non-exclusive, world-wide license to publish the Supplemental Material files. Please contact the corresponding author directly for reuse.
