# Supplementary material for: Heterosis of endophytic microbiomes in hybrid rice varieties improves seed germination
Source: mSystems. 2024 Apr 9;9(5):e00004-24. doi: 10.1128/msystems.00004-24 (PMC11097635; doi:10.1128/msystems.00004-24)
Supplement: Supplemental material — Fig. S1-S6; Tables S1 and S2. [file msystems.00004-24-s0001.pdf]

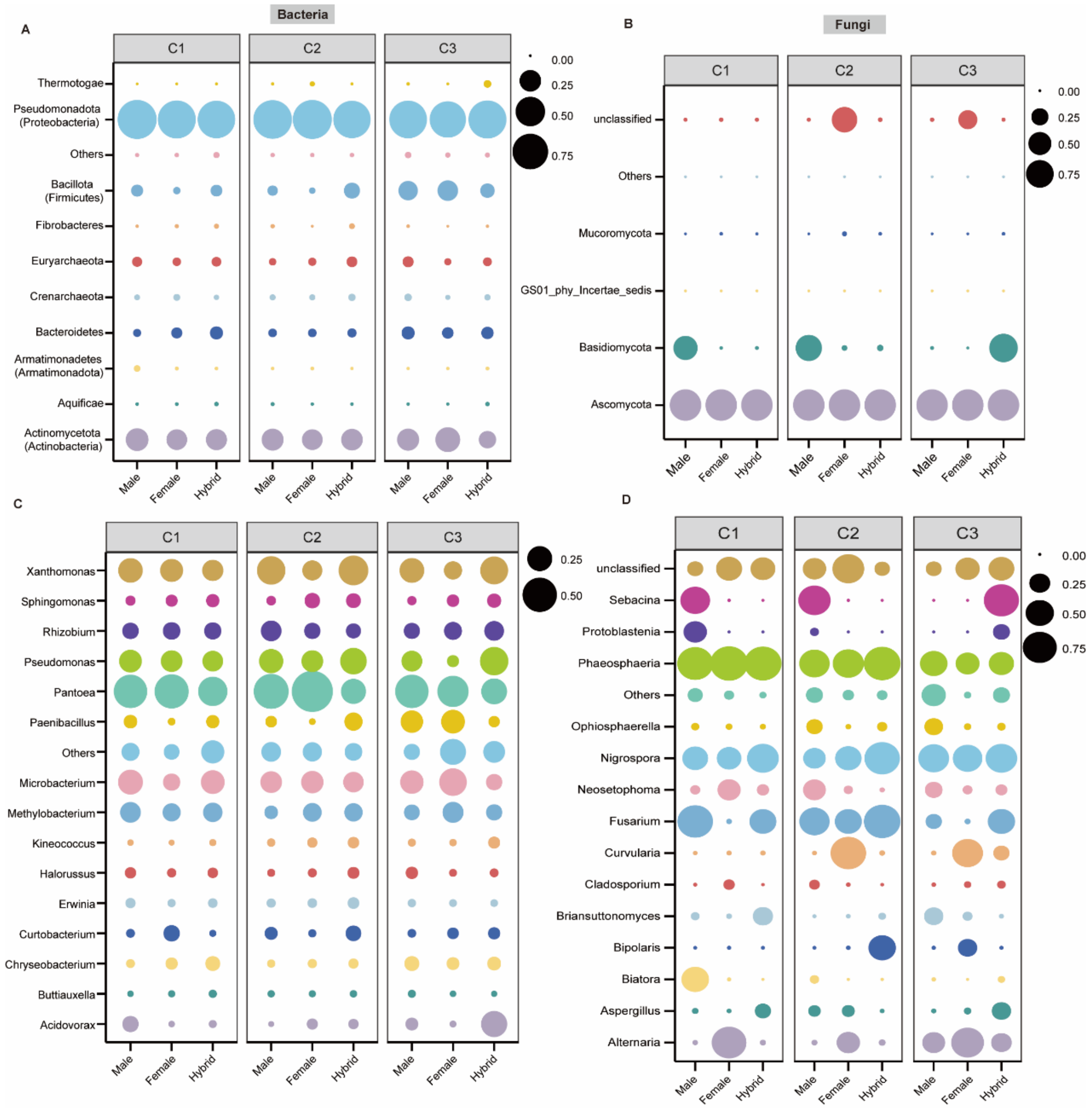

Fig. S1. Bubble plots showing the relative abundance of bacterial (A, C) and fungal (B, D) taxa at phylum and genus levels in seed from three hybrid variety combinations. C1 represents hybrid rice combination: HZ♂, TF♀ and TFHZ; C2 represents hybrid rice combination: H7♂, ZZA♀ and ZYH7; C3 represents hybrid rice combination: R261♂, HZA♀ and HZ261. Due to the updated bacterial classification names in 2021, the names in Figure A have been revised to reflect the updated nomenclature, while the old names are provided in parentheses. The size of bubble represents relative abundances of microbial community.

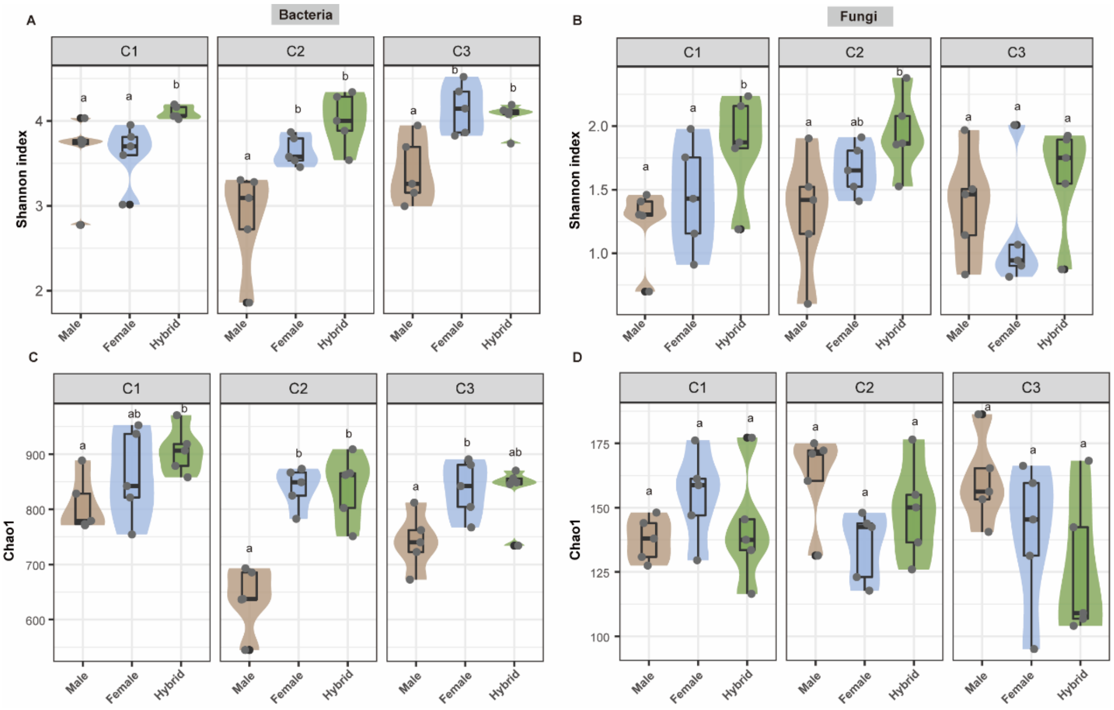

Fig. S2. Alpha diversity (Shannon and Chao1 indices) of seed endophytic bacterial (A, C) and fungal (B, D) communities in seed from three hybrid variety combinations. C1 represents hybrid rice combination: HZ♂, TF♀ and TFHZ; C2 represents hybrid rice combination: H7♂, ZZA♀ and ZYH7; C3 represents hybrid rice combination: R261♂, HZA♀ and HZ261. Different lowercase letters indicate a significant difference ($p < 0.05$; One-way ANOVA and Dunn's multiple-comparison test).

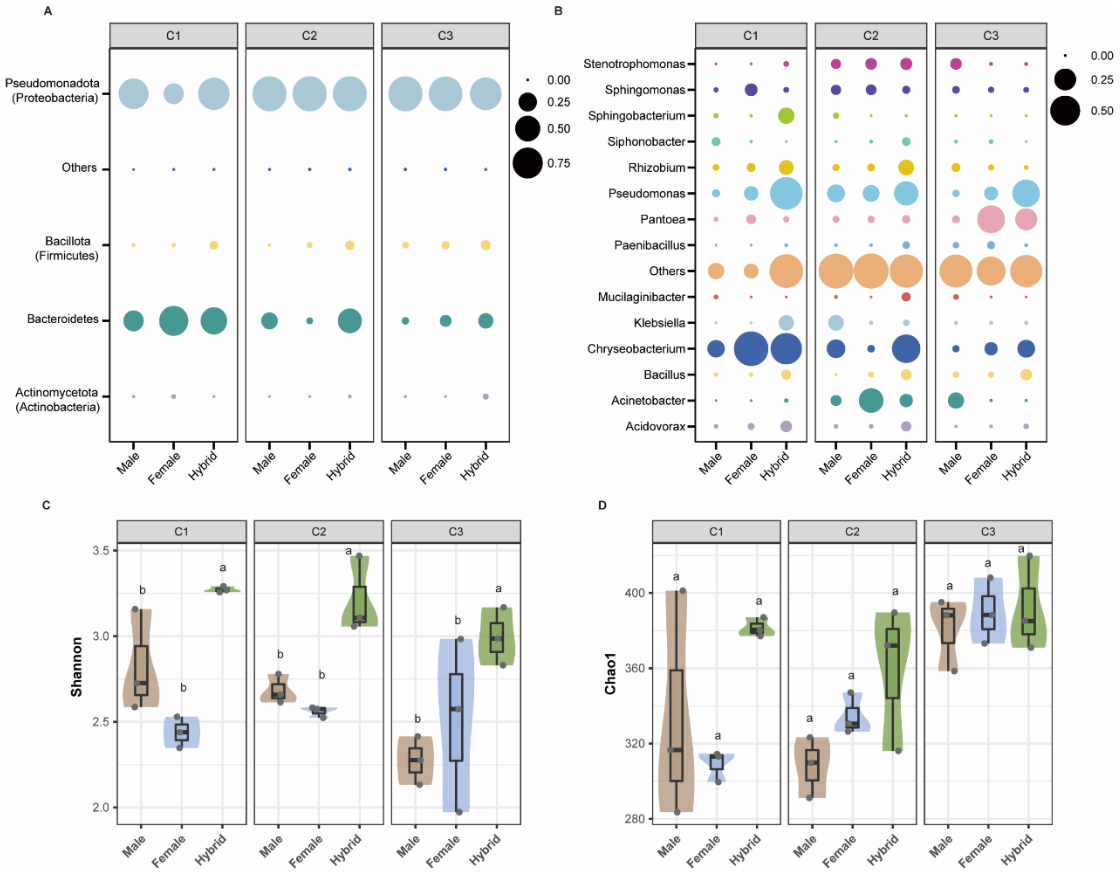

Fig. S3. The relative abundance of bacterial taxa at phylum and genus levels (A, B) and alpha diversity (Shannon and Chao1 indices) of culturable seed endophytic bacterial communities (C, D) in seeds from three hybrid variety combinations. C1 represents hybrid rice combination: HZ♂, TF♀ and TFHZ; C2 represents hybrid rice combination: H7♂, ZZA♀ and ZYH7; C3 represents hybrid rice combination: R261♂, HZA♀ and HZ261. Different lowercase letters indicate a significant difference ($p < 0.05$; One-way ANOVA and Dunn's multiple-comparison test).

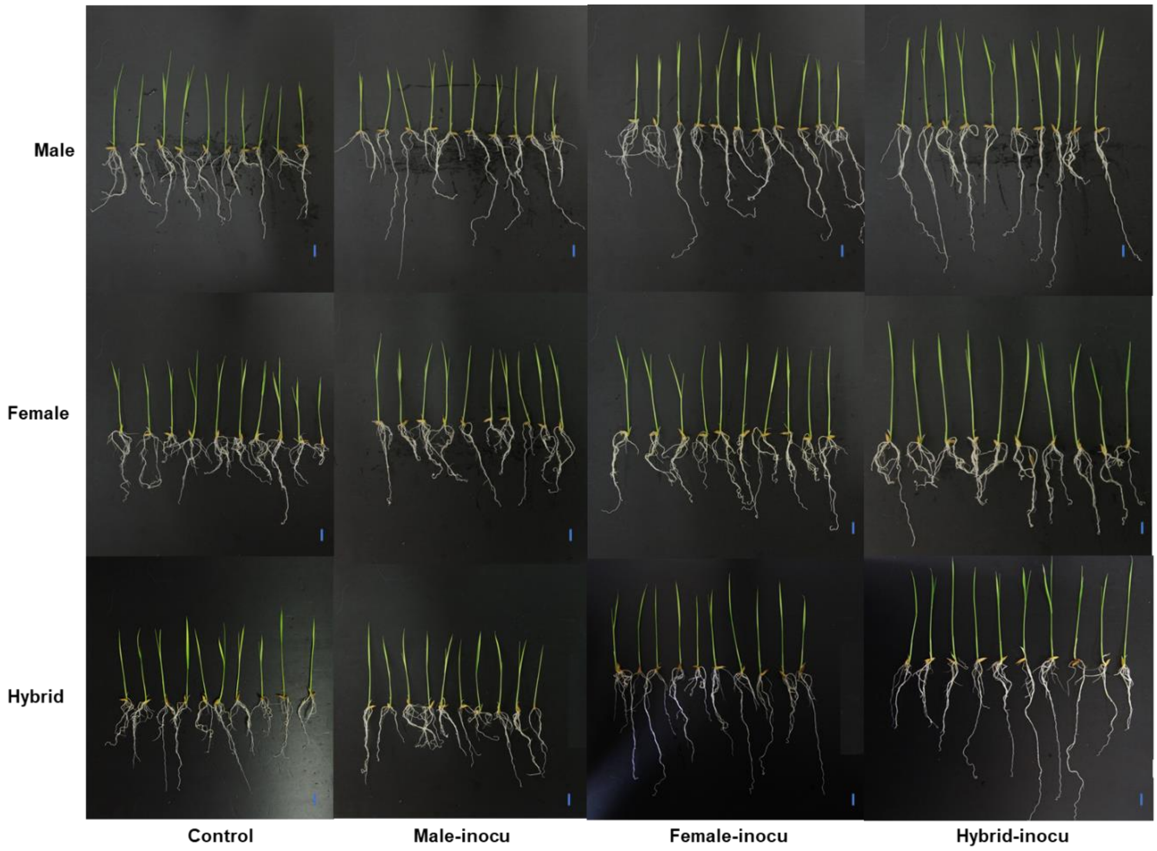

Fig. S4. The germination phenotype of seeds inoculated with seed endophyte suspensions (SES) obtained from both the hybrid and corresponding parental lines. Control: sterile phosphate-buffered saline (PBS) solution; Male-inocu, Female-inocu, and Hybrid-inocu represent seed endophytic microbiome suspensions enriched from male, female, and hybrid seeds, respectively. Bar: 1cm.

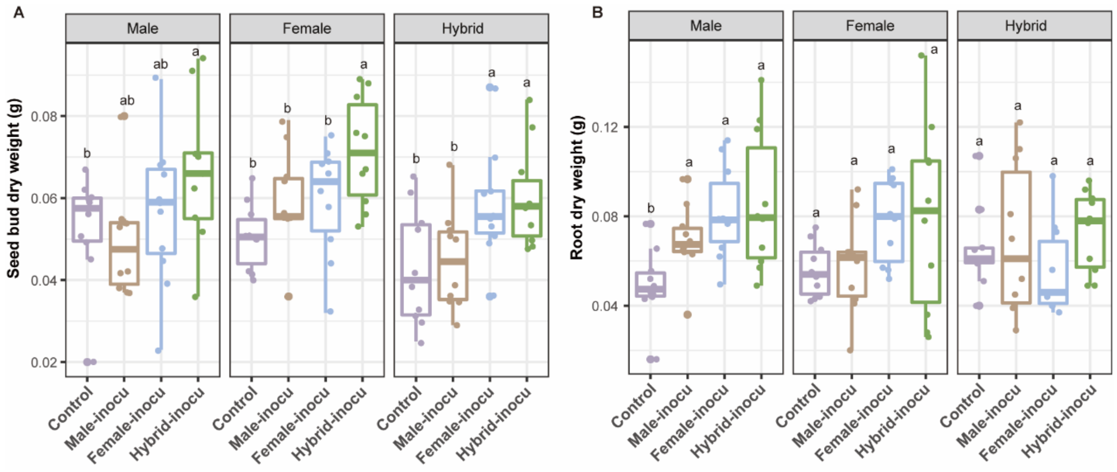

Fig. S5. The bud and root dry weight of seeds inoculated with seed endophytic microbiome suspensions. Different lowercase letters indicate a significant difference ($p < 0.05$; One-way ANOVA and Dunn's multiple-comparison test). Control: sterile phosphate-buffered saline (PBS) solution; Male-inocu, Female-inocu, and Hybrid-inocu represent seed endophytic microbiome suspensions enriched from male, female, and hybrid seeds, respectively.

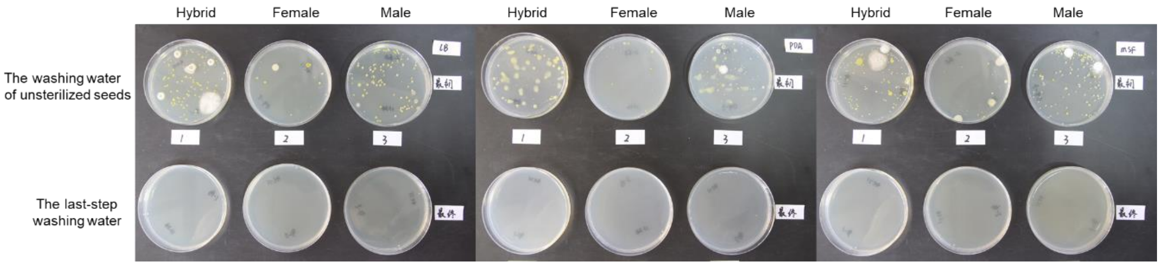

Fig. S6. The microbial growth of washing water from the last-step and unsterilized seeds in different growth agar-media. LB represents Luria Bertami media, MSF represents Mannitol Soya flour media and PDA represents Potato Dextrose Agar media.

Table S1. Topological properties of co-occurrence networks of seed endophytic microbial communities in different genotypes.

Topological properties	Male	Female	Hybrid
**Bacteria**			
Modularity	0.76	0.83	0.84
Number of vertices	270	260	257
Number of edges	934	700	742
Connectance	0.02	0.02	0.02
Average degree	6.91	5.38	5.77
Average path length	6.87	10.39	5.66
Diameter	18	31	16
Clustering coefficient	0.64	0.66	0.79
Number of clusters	8	14	18
Degree centralization	0.07	0.05	0.05
**Fungi**			
Modularity	0.84	0.78	0.75
Number of vertices	250	262	264
Number of edges	1990	2823	2584
Connectance	0.06	0.08	0.07
Average degree	15.92	21.54	19.57
Average path length	6.18	2.12	4.33
Diameter	16	8	16
Clustering coefficient	0.95	0.95	0.97
Number of clusters	14	13	15
Degree centralization	0.06	0.08	0.10

Table S2. Characteristics of rice genotypes selected for the microbiome analysis.

Denomination	Abbreviation	Category	Pedigree	Combination
Hua Zhan	HZ	Male	/	C1
Tian Feng A	TF	Female	/	C1
H7	H7	Male	/	C2
Zhong Zhe A	ZZA	Female	/	C2
R261	R261	Male	/	C3
Hua Zhe2A	HZA	Female	/	C3
Tianyouhuazhan	TFHZ	Hybrid	HZ♂×TF♀	C1
Zhongzheyou-H7	ZYH7	Hybrid	H7♂× ZZA♀	C2
Huazheyou-261	HZ261	Hybrid	R261♂×HZA♀	C3
